# Cases of Rheumatism

**Published:** 1826-07

**Authors:** 

**Affiliations:** the Westminster General Dispensary.


*Cases of Rheumatism.* Treated by Dr. Macleod, at the Westminster General Dispensary.

I. May 27th.—Jane Cripps, aged fifty-four, a washer-woman, residing in St. Martin's.

Complains of violent pain in both ankles, extending to the anterior part of the foot, and in the right wrist and hand: no swelling of the parts. Pains worse during the night; does not sweat. Pulse ninety-two; skin hot; bowels open from medicine. Has had pains flying about her for several days, but yesterday morning they fixed in her ankles, which at the same time became so stiff that she was unable to walk across the room. Has been subject for several years to occasional attacks of rheumatism.

℞. Hydrar. Submuriat. gr. x.; Opii, gr. ij. M. fiant pil. ij. omni nocte sumendæ.

Infus. Sennæ, ℥iss. cum Magnes. Sulphat. ʒij. omni mane.

30th.—Has taken two pills for three successive nights. Experienced no relief from the first. Was better yesterday, and is quite free from pain to-day. Bowels briskly purged; mouth sore.

Gargarisma aluminis. Omitt. alia.

June 1st.—Free from all complaint, except some remains of ptyalism.

Rep. gargarisma. Habeat Acid. Sulph. dil. gtt. x. ter die.

10th.—No return of pain; mouth quite well.

Omitt. omnia. Very good

II. June 3d.—Maria Fisher, aged twenty-five, residing in St. Ann's, Soho.

Complains of severe pain in the posterior part of the right leg, a little above the ankle-joint, where there is a diffuse swelling and some degree of redness. Slight pains in the arms; worse at night. Sweats copiously, without relief. Pulse 100; skin rather hot; tongue clean; bowels regular. Had a similar attack last year, which confined her for two months. At present has been ill for a week: attributes her complaints to exposure to cold. Took gtt. xxx. of the vinum colchici three times a-day, for three days during the present week, without any relief.

Hydrar. Submur. gr. x.; Opii, gr. ij. omni nocte.

Infus. Sennæ, ℥ss. c. Magnesiæ Sulphat. ʒij. omni mane.

6th.—Pains entirely gone. Mouth sore; bowels very open; occasional sickness and vomiting. Did not take the senna and salts.

Gargarisma aluminis. Omitt. medicamenta.

10th.—Cured.

It is now several years since the Editor adopted this method of treating acute rheumatism, in consequence of the success attending Dr. Chamber's practice at St. George's Hospital. The following case, which occurred in October 1820, and was mentioned at the time in this Journal, (vol. xliv. p. 437,) affords a striking illustration of its efficacy:—

III. A labourer, between thirty and forty, of robust constitution, was visited in the afternoon, when he was found in bed, with pains all over him, particularly in the wrists and ankles, which were red and swollen. He had much fever, and the slightest movement excited the greatest agony, so that he lay motionless upon his back. The attack had come on two days before, from exposure to cold. Two scruples of calomel and eight grains of opium were ordered to be made into eight pills, of which two were to be taken night and morning; senna and salts at mid-day.

On visiting him next day, he was found sitting by the fire, almost entirely free from pain, *having taken all the eight pills between six in the evening and ten next morning*. His mouth was made slightly sore, and he rapidly got well.

